# Use of Omics Technologies for the Detection of Colorectal Cancer Biomarkers

**DOI:** 10.3390/cancers14030817

**Published:** 2022-02-06

**Authors:** Marina Alorda-Clara, Margalida Torrens-Mas, Pere Miquel Morla-Barcelo, Toni Martinez-Bernabe, Jorge Sastre-Serra, Pilar Roca, Daniel Gabriel Pons, Jordi Oliver, Jose Reyes

**Affiliations:** 1 Grupo Multidisciplinar de Oncología Traslacional, Institut Universitari d’Investigació en Ciències de la Salut (IUNICS), Universitat de les Illes Balears, E-07122 Palma de Mallorca, Illes Balears, Spain; marina.alorda@uib.es (M.A.-C.); margalida.torrens@ssib.es (M.T.-M.); pere.morla@uib.es (P.M.M.-B.); toni.martinez@uib.es (T.M.-B.); jorge.sastre@uib.es (J.S.-S.); pilar.roca@uib.es (P.R.); d.pons@uib.es (D.G.P.); 2 Instituto de Investigación Sanitaria Illes Balears (IdISBa), Hospital Universitario Son Espases, Edificio S, E-07120 Palma de Mallorca, Illes Balears, Spain; 3 Translational Research in Aging and Longevity (TRIAL) Group, Instituto de Investigación Sanitaria Illes Balears (IdISBa), E-07120 Palma de Mallorca, Illes Balears, Spain; 4 Ciber Fisiopatología Obesidad y Nutrición (CB06/03) Instituto Salud Carlos III, E-28029 Madrid, Madrid, Spain; 5 Servicio Aparato Digestivo, Hospital Comarcal de Inca, E-07300 Inca, Illes Balears, Spain

**Keywords:** omics, colorectal cancer, extracellular vesicles, tumour tissue, blood, stool, bowel lavage fluid, urine, breath

## Abstract

**Simple Summary:**

Colorectal cancer (CRC) is one of the most frequent cancers worldwide. Early detection of CRC is crucial, as it greatly improves the survival of patients. Currently, the CRC screening programs consist of a stool test to detect the presence of blood in stool and a subsequent colonoscopy to confirm the diagnosis. However, CRC screening can be further improved with the use of new biomarkers. Omics technologies, that is, techniques that generate a vast amount of data, can help to establish these markers. Here, we discuss the use of omics with different types of samples (breath, urine, stool, blood, bowel lavage fluid, and tissue) and highlight some of the most relevant biomarkers that have been recently detected.

**Abstract:**

Colorectal cancer (CRC) is one of the most frequently diagnosed cancers with high mortality rates, especially when detected at later stages. Early detection of CRC can substantially raise the 5-year survival rate of patients, and different efforts are being put into developing enhanced CRC screening programs. Currently, the faecal immunochemical test with a follow-up colonoscopy is being implemented for CRC screening. However, there is still a medical need to describe biomarkers that help with CRC detection and monitor CRC patients. The use of omics techniques holds promise to detect new biomarkers for CRC. In this review, we discuss the use of omics in different types of samples, including breath, urine, stool, blood, bowel lavage fluid, or tumour tissue, and highlight some of the biomarkers that have been recently described with omics data. Finally, we also review the use of extracellular vesicles as an improved and promising instrument for biomarker detection.

## 1. Introduction

Colorectal cancer (CRC) is one of the most frequently diagnosed cancers, with more than 1.9 million estimated new cases worldwide [1]. In Spain, CRC accounted for around 15,288 deaths in 2018, and has an annual age-standardized mortality rate of 30 per 100,000 inhabitants. This makes CRC the sixth-leading cause of death and the second leading cause of cancer-related mortality [2]. Early diagnosis raises the 5-year survival rate of these patients up to 94% [3]. Given the high burden of CRC on the National Health Service and the importance of early detection, significant efforts have been directed toward developing CRC screening programs. The main aim of these programs is to remove pre-malignant lesions which could ultimately develop into malignant tumours, as well as to start treatment in early-stage detected cancers. This way, it is expected to reduce CRC incidence and CRC-specific mortality, which has been proven effective [4].

One of the main problems for CRC is the late diagnosis, giving rise to a decrease in survival since there is a lack of early biomarkers [5]. Different tools have been developed for CRC screening, which include colonoscopy, flexible sigmoidoscopy, guaiac faecal occult blood testing (gFOBT), faecal immunochemical testing (FIT), and carcinoembryonic antigen (CEA) in plasma, which has low sensitivity and specificity [6]. Intention-to-treat estimates from meta-analyses of large randomized trials report reductions in CRC mortality of 20–30% for flexible sigmoidoscopy [7,8], 8–16% for gFOBT, and 41% for FIT and follow-up colonoscopy [9]. Currently, the screening program in Spain consists of biennial FIT with colonoscopy follow-up on positive subjects, according to the European guidelines [10]. However, every autonomous region implements this program at a different pace and there are important differences among regions [11,12]. Although this screening program has led to a decrease in mortality, the performance of this test is suboptimal, with a sensitivity and specificity for CRC of 54–89% and 89–97%, respectively [13]. Furthermore, it has been noted that this sensitivity may vary with the tumour stage, being lower with early-stage CRC [14]. This leads to a substantial number of false negative and false positive tests and, consequently, to missed diagnoses or unneeded colonoscopies. Thus, there is an urgent need for more accurate and, ideally, non-invasive tests to implement for CRC screening and monitoring tumour progression and treatment efficacy. 

The emergence of omics technologies is a promising strategy for detecting biomarkers of CRC. These methods generate high-throughput data that have the potential to detect significant changes that reflect the tumour initiation and progression. In this review, we discuss the utility of these technologies in different types of samples, such as breath, urine, stool, blood, bowel lavage fluid, and tumour tissue, and highlight some of the most promising results obtained in recent years. Finally, we also consider the isolation of extracellular vesicles (EVs) as an enhanced tool to detect new disease biomarkers. 

## 2. Omics Techniques

### 2.1. Genomics

The National Cancer Institute defines genomics as the study of the complete set of DNA (including all of its genes) in a person or other organism. The genome contains all the information needed for an individual to develop and grow. Analyzing the genome may help researchers understand how genes interact with each other and the environment and how certain diseases, such as cancer, diabetes, or heart disease develop. This may lead to new ways to diagnose, treat, and prevent disease [15]. Genetic alterations have been identified as major players in tumourigenesis. Therefore, genomics has gained attention as a tool to identify genetic markers that can lead to better diagnosis and prognosis and at the same time, allow researchers to improve the understanding of cancer. Apart from gene mutations and single nucleotide polymorphisms (SNP), the epigenetic signature has also proven useful to establish a more personalised diagnosis [16]. 

The development of high-throughput methods for genome and gene expression studies has increased the amount of information available. These data are deposited in international public repositories and can be studied by other research groups. NCBI Gene Expression Omnibus (GEO) is the most important database repository of high-throughput gene expression data and hybridization arrays, chips, and microarrays [17]. The Cancer Genome Atlas (TCGA) of the National Cancer Institute (NCI) is another relevant database in oncology. TCGA is a project to classify the genetic mutations that cause cancer, using genome sequencing and integrating bioinformatics tools to analyse this information [18].

Finally, the use of metagenomics, which evaluates the microbiome genes, holds special promise for CRC. Metagenomics has shown the potential to identify differences between control and CRC-associated microbiomes and eventually describe new CRC biomarkers [19].

### 2.2. Transcriptomics

Transcriptomics is the study of all RNA molecules in a cell and could give more information about how genes are turned on and off in different cell types and how this can contribute to cancer [20]. Differential gene expression comparison studies have emerged as a prospective approach to detecting promising biomarkers of enormous clinical value. This type of study is fuelled by and analyses the data deposited in the TGCA and GEO databases [21].

### 2.3. Proteomics

Proteomics is the study of the structure and function of proteins, including how they work and interact with each other [22]. In the search for new CRC biomarkers, proteomics studies are focused on differential protein expression between normal and cancer cells or the detection of different proteomic profiles in corporal fluids. Some of the most useful techniques for the identification of protein biomarkers in cancer are two-dimensional gel electrophoresis coupled with liquid chromatography/mass spectrometry (2-DE-MS), two-dimensional difference gel electrophoresis (2D-DIGE), or liquid chromatography–mass spectrometry (LC-MS) [23]. Multiplexed quantitative proteomic assays are capable of measuring changes in proteins and their interacting partners, isoforms, and post-translational modifications [23].

### 2.4. Metabolomics

Metabolomics is the study of metabolites in cells and tissues, which can be measured in different body fluids. The presence of a tumour can alter the whole individual’s metabolism, and the use of some fuels can be modified to meet the energy demands of the tumour. Furthermore, the tumour metabolism may change as the tumour progresses. Considering that the dysregulation of metabolism is one of the hallmarks of cancer, this omics could open a new way to study cancer [24].

### 2.5. Glycomics

Glycomics studies the structure and function of glycans, N- and O- linked glycoproteins, glycolipids, and proteoglycans [25,26]. The most common alterations in lipid and protein glycosylation are an increase in the branching of N-glycans, high density of O-glycans, incomplete glycans synthesis, neosynthesis, and sialylation and fucosylation increase [27]. Glycans characterization can be done by a large number of techniques, such as microarrays, flow cytometry, enzyme-linked immunosorbent assay, mass spectrometry, and chromatographic techniques [27].

### 2.6. Volatolomics

Volatolomics is the study of volatile organic compounds that have high vapor pressure. This is a non-invasive, fast, and potentially inexpensive way of analysing the human body chemistry for monitoring of diseases such as cancer [28]. The volatilome, volatile organic compounds (VOC) profile, is being used in the detection of CRC. Alterations in the metabolism of cancer cells can be reflected in a characteristic profile of VOCs, as these compounds are produced in metabolic processes such as inflammation, cancer metabolic alterations, and necrosis processes [29,30,31,32,33]. Cancer-associated VOCs are directly excreted from the affected organ or tissue to stool or blood. Thus, the VOCs are exhaled in breath, excreted in urine, or released from the skin [34,35,36]. However, the VOCs interactions with the microbiota may affect the volatilome of stool [29]. The most used techniques in volatolomics are gas chromatography with mass spectrometry (GC–MS), which enables the separation and quantification of individual VOCs; proton transfer reaction—mass spectrometry (PTR-MS), for simultaneous real-time monitoring of VOCs without sample preparation; and eNose, which allows the analysis of a specific VOC pattern in real-time. The latter is a low cost, easy-to-use equipment that can detect cancer at an early stage and can differentiate between cancer and healthy subjects [29,33,37]. 

Several studies have demonstrated the potential of the exhaled volatilome for CRC diagnosis and screening due to its sensitivity and specificity. However, further studies and standardization of collection and analysis methods for volatilome detection and its application to CRC diagnosis are needed [31,35,36,37,38,39].

## 3. Sample Types for the Omics Analyses in Colorectal Cancer

### 3.1. Breath Samples 

Breath is a type of non-invasive sample easily collected that can be used to diagnose CRC. 

#### Volatolomics

The determination of the volatilome in breath could provide new biomarkers for the detection of CRC. De Vietro et al. have shown differences in the release of VOCs between normal and cancerous colonic mucosa, the latter releasing higher amounts of benzaldehyde, benzene ethyl, and indole; these compounds can be detected in the breath of patients [30]. Politi and collaborators analysed the VOCs of different types of cancers, and specifically reported dinitrogen oxide, nitrous acid, acetic acid, and 1,3-butadiene in the breath of CRC patients [32].

Haick and Hakim have patented a colon cancer VOC marker, 1,3,5-cycloheptatriene. This compound is present in the breath of CRC patients and is not found in other types of cancer (breast, prostate, head, and neck cancer) or in healthy subjects. Moreover, other compounds can be found in the breath of these patients, such as 1,1′-(1-butenylidene) bis benzene, 1methyl-3-(1-methylethyl) benzene, 1-iodo nonane, [(1,1-dimethylethyl) thio] acetic acid, 2-amino-5-isopropyl-8-methyl-1-azulenecarbonitrile, 3,3-dimethyl hexane, 1-ethyl-2,4-dimethyl benzene, 1,1′-(3-methyl-1-propone-1,3-diyl)bis benzene, 2-methyl 1,3,butadiene. However, several of these compounds are also found in other types of cancers or healthy subjects [40].

Recent studies using discriminatory models with 14 VOCs (see Table 1) exhaled by patients were able to discriminate between patients with CRC and healthy patients. These models had a statistically significant likelihood of discrimination with an area under the ROC curve of 0.979 [41].

### 3.2. Urine Samples

Urine is a sample that can be easily collected and is a non-invasive method for detecting molecules related to CRC, such as blood and stool.

#### 3.2.1. Genomics

The latest advances in urine genomics focus on the study of mutations in KRAS. Ohta et al., evaluated the quantity of ctDNA derived from urine (transrenal ctDNA) and the accuracy of *KRAS* mutation detection in relation to CRC stage [42].

#### 3.2.2. Proteomics 

The urine of patient-derived xenograft (PDX) mice with CRC tumours has been evaluated to find protein biomarkers [43]. This approach has helped to improve the clinical efficacy of markers of colorectal liver metastasis, such as CEA [44]. Moreover, the cargo of exosomes as a source for proteomics studies has been recently studied, not only in urological cancers, but also in non-urological cancers such as CRC. Erozenci and collaborators analysed MS-based proteomic data on urinary exosomes from cancer patients and discussed the potential of urinary exosome-derived biomarkers in cancer [45].

#### 3.2.3. Metabolomics 

In a systematic review, up to 244 compounds in urine samples from cancer patients were identified [46]. Four upregulated metabolites and seven downregulated compounds were reported in the metabolome and the volatilome, as shown in Table 2 [46].

Interestingly, in one study comparing the metabolic profile of plasma, stool, and urine of advanced colon cancer and healthy subjects, the authors determined that metabolites from the stool samples were negatively correlated with those found in the urine samples [47]. In another study, 154 metabolites were identified, including metabolites of glycolysis, tricarboxylic acid (TCA) cycle, amino acids, urea cycle, and polyamine pathways. The concentration of these metabolites gradually increased with the stage of cancer, with the difference in stage IV being the greatest. Furthermore, the analysis of metabolites allowed for discriminating between polyps and CRC samples [48]. Ning et al. described eleven metabolites that were up-regulated, while four other metabolites were down-regulated in urine samples from CRC patients compared to healthy controls, as shown in Table 2. Analysing the pathways involving these metabolites, they found alterations in the energy metabolism in CRC patients, reflecting an upregulation of glycolysis and amino acid metabolism and a decrease in lipid metabolism [49].

On the other hand, a new metabolomics-based urine test (UMT) can detect adenomatous polyps and CRC. According to the authors, this UMT could be more cost-effective if used in CRC screening programs [50]. Another approach is urine nuclear magnetic resonance (NMR) metabolomics as a diagnostic tool for early detection of CRC [51]. 

Other studies have also been developed with a focus on the diet, specifically with the presence of metabolites derived from white beans. Concretely, a dietary intervention was carried out for 4 weeks with white beans, and changes in different metabolic pathways which are important for CRC prevention were observed [52]. All biomarkers are summarized in the Table 2.

**Table 2:** Main biomarkers found in urine samples of CRC patients with metabolomics.

Omics	Biomarker	Change	Reference
Metabolomics	3-hydroxybutyric acid, L-dopa, L-histidinol, and N1, N12-diacetylspermine	Upregulated	[46]
Metabolomics	pyruvic acid, hydroquinone, tartaric acid, hippuric acid, butyraldehyde, ether, and 1,1,6-trimethyl-1,2-dihydronaphthalene	Downregulated	[46]
Metabolomics	Hydroxyproline dipeptide, tyrosine, glucuronic acid, tryptophan, pseudouridine, glucose, glycine, histidine, 5-oxoproline, isocitric acid, threonic acid	Upregulated	[49]
Metabolomics	Citric acid, octadecanoic acid, hexadecanoic acid, propanoic acid-2-methyl-1-(1,1-dimethylethyl)-2-methyl-1,3-propanediyl ester	Downregulated	[49]
Metabolomics	3-(4-hydroxyphenyl)propionate, betaine, pipecolate, S-methylcysteine, choline, eicosapentaenoate (20:5n3), benzoate, S-adenosylhomocysteine, N-delta-acetylornithine, cysteine, 3-(4-hydroxyphenyl)lactate, gentisate, hippurate, 4-hydroxyhippurate, and salicylate.	Up- and downregulated	[52]

### 3.3. Stool Samples

The use of stool samples offers several advantages as a source of CRC biomarkers. Sample collection is non-invasive, the test can be performed at home, there is no sample amount limitation, and the stool effectively samples the entire length of the inner bowel wall contents (including tumour) as it passes down the gastrointestinal tract [53]. For this reason, stool samples are increasingly gaining attention in the search for new biomarkers for the early detection of CRC [54,55].

#### 3.3.1. Genomics 

In one study analysing the stool microbiome, four gene markers were identified to be enriched in early-stage (I-II) CRC patients, highlighting the potential for using stool metagenomic biomarkers for the early diagnosis of CRC [19]. Among these four genes, butyryl-CoA dehydrogenase from *F. nucleatum* was identified as the best potential CRC biomarker [19]. Another study has shown an increase of gut microbial *baiF* gene copy numbers in CRC patients’ stool samples, in addition to *baiF* RNA expression [56]. In another interesting study, the authors compared the gut microbiome between CRC patients and their healthy family members, to avoid lifestyle interferences, by sequencing extracted DNA from stool samples. The best biomarker they obtained was from *Coprobacillus* [57]. In a very recent study with more than 1,000 participants, a metagenomics analysis was carried out and results were validated by targeted quantitative PCR. The authors identified a novel bacterial marker, *m3*, from *Lachnoclostridium* species for adenoma detection [58]. 

On the other hand, the utility of DNA methylation as a biomarker for CRC has been analysed. One study identified 4 potential methylation markers (*COL4A1*, *COL4A2*, *TLX2*, and *ITGA4*) upregulated in CRC patients’ stool, using real-time methylation-specific PCR based on TaqMan probe fluorescence (TaqMan qMSP) technology after a selection of these genes in CRC cell lines and CRC patients’ tissue [59]. Another similar report performed a methylation analysis using MethyLight qPCR and droplet digital PCR, reporting elevated CpG islands methylation in two genes: *GRIA4* and *VIPR2* [60]. Two additional DNA methylation markers analysed with multiplex quantitative PCR assay, *SDC2* and *NDRG4*, were found in another study, providing a solid foundation for multi-target DNA biomarker analysis in stool samples for CRC screening [61]. Previously, other authors reported *SDC2* methylation as a good candidate for potential non-invasive diagnostic tool for early detection of CRC [62]. In fact, a clinical trial was conducted in 2020, with more than 1000 participants to assess a stool DNA test of methylated *SDC2* for CRC detection, with promising results [63]. In a study published in September 2021, *SDC2* methylation, as well as *ADHFE1* and *PPP2R5C* methylation, have been revealed as good CRC biomarkers, confirming the accuracy of *SDC2* methylation as a CRC indicator [64]. Another gene promoter methylation, *SOX21*, was demonstrated in a very recent analysis in stool to be a good non-invasive biomarker with a high sensitivity and specificity [65]. All these biomarkers are summarized in Table 3.

#### 3.3.2. Transcriptomics

Almost 15 years ago, the very first studies for the search and standardization of transcriptomic molecular markers in CRC patients’ stool were conducted, as the authors assured that RNA-based detection methods were more comprehensive than either DNA-, protein- or methylation-based screening methods [66]. Years later, an analysis of miRNAs in the stool of control and CRC patients was performed [67]. In this study, seven miRNAs were found to be upregulated in CRC (miR-21, miR-106a, miR-96, miR-203, miR-20a, miR-326 and miR-92) and another seven were found to be downregulated (miR-320, miR-126, miR-484-5p, miR-143, miR-145, miR-16 and miR-125b) [67]. Interestingly, the authors correlated miRNAs with their target mRNAs, having a more precise idea of the activated or inhibited molecular pathways [67]. In a more recent study, the same authors revealed 12 miRNAs (miR-7, miR-17, miR-20a, miR-21, miR-92a, miR-96, miR-106a, miR-134, miR-183, miR-196a, miR-199a-3p, and miR-214) overexpressed and 8 miRNAs (miR-9, miR-29b, miR-127-5p, miR-138, miR-143, miR-146a, miR-222, and miR-938) with decreased expression in CRC patients’ stool [68]. The novelty of this study is the fact that these changes in expression were more pronounced as the cancer progressed from early to late TNM stages [68]. Another type of RNAs, lncRNAs, were recently used to do a panel of potential biomarkers for early detection of CRC in stool colonocytes, including CCAT1, CCAT2, H19, HOTAIR, HULC, MALAT1, PCAT1, MEG3, PTENP1, and TUSC7 [69]. All biomarkers are summarized in Table 3.

#### 3.3.3. Proteomics 

In recent years, several studies have investigated new methods to mine deeply into the stool proteome to reveal candidate proteins to be potential biomarkers for CRC [53]. A recent publication has reviewed the main biomarkers obtained in stool samples among other corporal fluids and biopsies [70]. Using tandem mass spectrometry (LC-MS/MS), Yang et al. identified seven proteins (Hp, LAMP1, SYNE2, LRG1, RBP4, FN1, and ANXA6) alone or in combination, to detect high-risk adenomas and CRCs [71]. All biomarkers are summarized in Table 3.

#### 3.3.4. Metabolomics 

In a study carried out in China, GC-MS based metabolomics approach was used to discriminate healthy individuals from CRC patients, associating different metabolites with health status or disease phenotype. In this work, polyamines (cadaverine and putrescine) were found as potential biomarkers for CRC prediction [72]. Previously, in a clinical trial done in Korea, the same method revealed changes in the fatty acid metabolome of CRC patients, implying that stool fatty acids, concretely increased oleic acid, could be used as a novel screening tool for CRC [73]. Differences in cholesteryl esters and sphingolipids have also been found in the stool of CRC patients using an UHPLC-MS metabolomics approach [74]. Recently, using the proton nuclear magnetic resonance (^1^H NMR) technique, downregulation of butyrate and upregulation of alanine, lactate, glutamate and succinate was reported in CRC tissue and stool [75]. Finally, it is important to note the relationship between the metabolomic profile and the microbiota presence in the stool. Some studies have shown changes in the microbiome, such an enrichment of *Proteobacteria*, *Fusobacteria, Parvimonas,* and *Staphylococcus* in CRC and *Firmicutes* in healthy groups as well as an uneven and lesser microbial diversity in CRC [72,74]. All biomarkers are summarized in Table 3.

**Table 3:** Main biomarkers found in stool samples of CRC patients with different omics technologies.

Omics	Biomarker	Change	Reference
Genomics (metagenomics)	butyryl-CoA dehydrogenase from *F. nucleatum*	Upregulated	[19]
Genomics and Transcriptomics	*baiF*	Upregulated	[56]
Genomics (metagenomics)	*Coprobacillus*	Upregulated	[57]
Genomics (metagenomics)	*m3* from *Lachnoclostridium*	Upregulated	[58]
Genomics (methylation)	*COL4A1, COL4A2, TLX2, ITGA4*	Upregulated	[59]
Genomics (methylation)	*GRIA4, VIPR2*	Upregulated	[60]
Genomics (methylation)	*SDC2, NDRG4*	Upregulated	[61]
Genomics (methylation)	*SDC2*	Upregulated	[62]
Genomics (methylation)	*SDC2, ADHFE1, PPP2R5C*	Upregulated	[64]
Genomics (methylation)	*SOX21*	Upregulated	[65]
Transcriptomics (miRNAs)	miR-21, miR-106a, miR-96, miR-203, miR-20a, miR-326, miR-92	Upregulated	[67]
Transcriptomics (miRNAs)	miR-320, miR-126, miR-484-5p, miR143, miR-145, miR-16, miR-125b	Downregulated	[67]
Transcriptomics (miRNAs)	miR-7, miR-17, miR-20a, miR-21, miR-92a, miR-96, miR-106a, miR-134, miR-183, miR-196a, miR-199a-3p, miR-214	Upregulated	[68]
Transcriptomics (miRNAs)	miR-9, miR-29b, miR-127-5p, miR-138, miR-143, miR-146a, miR-222, miR-938	Downregulated	[68]
Transcriptomics (lncRNAs)	CCAT1, CCAT2, H19, HOTAIR, HULC, MALAT1, PCAT1, MEG3, PTENP1, TUSC7	Upregulated	[69]
Proteomics	Hp, LAMP1, SYNE2, LRG1, RBP4, FN1, ANXA6	Upregulated	[71]
Metabolomics	Polyamines (cadaverine and putrescine)	Upregulated	[72]
Metabolomics	Cholesteryl esters, Sphingomyelins	Upregulated	[74]
Metabolomics	Oleic acid	Upregulated	[73]
Metabolomics	Butyrate, Alanine, Lactate, Glutamate, Succinate	Upregulated (except Butyrate downregulated)	[75]

### 3.4. Blood Samples 

In recent years, the low invasiveness and easy accessibility of liquid biopsies have made them the object of many studies, since the biologic materials present in blood samples are potential sources of non-invasive biomarkers that could improve CRC diagnosis and prognosis [76].

Detection of serum or plasma CRC biomarkers from blood samples is challenging due to their low concentration and the presence of material from healthy cells. However, the recent development of separation methods and sample processing has improved the analysis [77]. In blood, we can identify circulating free DNA or RNA (cfDNA and cfRNA). These cfDNA and cfRNA can be circulating tumour DNA or RNA (ctDNA and ctRNA), which can come from tumour cells, tumour circulating cells (CTCs), and EVs. Complementarily, different proteins and metabolites released into the blood circulation can be detected [78].

#### 3.4.1. Genomics

Recent studies have focused on cfDNA as a quite important biomarker [79,80,81]. Quantification of total cfDNA and the DNA integrity index (DII: ratio of long DNA fragments resulting from necrosis and short DNA fragments resulting from apoptosis) have been reported elevated in CRC patients by using ALU and GAPDH sequences [80,81]. Furthermore, cfDNA are highly influenced by tumour stage and chemotherapy treatment; it is possible to also analyse point mutations, hypermethylation of gene promoters, or microsatellite alterations or MSI [76]. In fact, metastatic CRC presents less fragmented cfDNA compared to primary CRC [81]. In addition, KRAS, APC, and TP53 are the most featured genes with mutations after the analysis of the Idylla panel (KRAS, NRAS, and BRAF mutations, and characterization of MSI), the PlasmaSELECT-R panel (sequence alterations and translocations in 63 genes), the Guardant360 panel (point mutations in 70 genes and identification of gene fusions, insertions and deletions), the OncoBEAM panel (CRC-specific mutations), and the MassDetect CRC panel [78,82]. Furthermore, MSI is detected in 15% of CRCs and associated with defects in DNA mismatch repair genes [83]. and with a greater resistance to chemotherapy [84]. Despite being a marker that can be detected in 35% of CRC patients, the detection rate varies from 0 to 60% in studies [76].

Finally, it is important to consider the epigenetic signature. The use of different commercial tests, such as Epi proColon 2.0 (Epigenomics) or RealTime ms9 (Abbptt), has identified various hypermethylation sites (BCAT1 and IKZF1) [78] and aberrant methylation in numerous genes (APC, MLH1, FRP2, NGFR) and gene promoters [76,78]. Nonetheless, the most promising potential epigenetic biomarker is the SEPT9 gene, which has shown 90% of sensitivity and 88% of specificity [76,78]. All biomarkers are summarized in Table 4. 

#### 3.4.2. Transcriptomics 

The detection of differential gene expression could be influenced by the stages of CRC [76,78,85]. Most of these studies found differential expression of CK19, CK20, and CEA, whose overall sensitivity was up to 72% [85]. Moreover, studies based on expression panels identified *MDM2, DUSP6, CPEB4, MMD, EIF2S3*, *ANXA3*, *CLEC4D*, *LMNB1*, *PRRG4*, *TNFAIP6*, *VNN1*, and *IL2RB* as differentially expressed genes [76,78].

On the other hand, some of the most relevant miRNA molecules assessed, such as miR-145, miR-143, miR-135, and miR-17-92, showed a significant diagnostic value for advanced neoplasia [86]. Furthermore, a recent study has used SHERLOCK-based miRNA detection to enhance and facilitate exosome-miRNA detection in blood, showing miR-126, miR-1290, miR-23a, and miR-940 as the best predictive biomarkers for early CRC stages [87]. Other miRNAs have been described as potential biomarkers, such as miR-92a, miR-29a, miR-125b, miR-19a-3p, miR-223–3p, miR-92a-3p and miR-422a, although miR-21 is by far the most studied for CRC [78]. Currently, there are few studies about circulating lncRNA as a potential non-invasive diagnostic biomarker in CRC. In fact, only three transcripts (CRNDE-h, CCAT1 and HOTAIR1) are described as promising biomarkers [76,78]. However, UCA1 and circHIPK3 are lncRNA from serum exosomes which could discriminate against CRC [78]. All biomarkers are summarized in Table 4. 

#### 3.4.3. Proteomics

Currently, CEA and Carbohydrate Antigen 19-9 (CA19-9) are the standard biomarkers used for monitoring CRC patients using blood-based tests. Elevated CEA and CA19-9 levels correlate with poor CRC prognosis. However, increased CEA levels are not exclusive to CRC, as its increase can also be associated with other diseases, such as intestinal inflammation, pancreatitis, liver disease and other malignancies. For this reason, although this biomarker is more specific and sensitive than the CA19-9 antigen, there is a need to increase the panel of biomarkers to improve CRC diagnosis [76]. Consequently, Giessen et al. improved the specificity of CEA by combining it with serum amyloid A (SAA) [88].

Chen and collaborators demonstrated that Mammalian STE20-like protein kinase 1/Serine threonine kinase 4 (MST1/STK4), S100 calcium-binding protein A9 (S100A9) and tissue inhibitor of metalloproteinases 1 (TIMP1) can be used as CRC biomarkers [83]. In addition, a high correlation of Cysteine-rich 61 (Cyr61) with advanced CRC stages has been described [89]. In this context, Bhardwaj et al. reported a potential panel based on 12 proteins for the early detection of CRC [90].

Additionally, blood antibodies produced in response to tumour-associated antigens (TAAs) have been studied. The panel proposed by Villar-Vázquez et al. focuses on general transcription factor IIB (GTF2B), EGF-like repeats, discoidin I-like domains 3 (EDIL3), HCK, PIM-1, STK4 and tumour protein P53 [91]. All biomarkers are summarized in Table 4. 

#### 3.4.4. Metabolomics

The study of blood-related metabolites as potential non-invasive biomarkers of CRC has increased in the recent years [92]. The comparison between healthy individuals and CRC patients revealed a decrease in serum glucose levels, as well as lower concentration of novel circulating long-chain hydroxy fatty acids, especially GTA-446 [93,94]. On the other hand, the activation of glycolysis and glycine, serine, and threonine metabolism was observed by Gu et al. through CRC serum ^1^H-NMR analysis, reflecting the rapid consumption of energy due to the Warburg effect [95]. Furthermore, Nishiumi et al. developed a preliminary but potential panel based on 8 metabolites (pyruvic acid, tryptophan, lysine, glycolic acid, palmitoleic acid, ornithine, fumaric acid, and 3-hydroxyisovaleric acid) for early detection of CRC in plasma [96]. All biomarkers are summarized in Table 4. 

#### 3.4.5. Glycomics

The study of plasma IgG glycans presented some alterations that are associated with CRC mortality, such as a decrease in galactosylation and sialylation of fucosylated IgG glycan structures, in addition to an increase in bisecting GlcNAc in IgG glycan structures [97]. Furthermore, Doherty and collaborators found several glycome alterations in plasma associated with CRC: increase of glycans with no galactose residues (agalactosylation), decrease of mono- and di-galactosylated structures, increase of tri- and tetra-galactosylated glycans (galactosylation), decrease of mono-sialyted glycans and increase of tri- and tetra-sialyted structures (sialylation), decrease of galactosylated and sialylated bi-antennary GlcNAc glycans, increase of highly branched glycans (GlcNAc antennae) and decrease of neutral core fucosylated glycans with one or two galactose residues (core fucose) [98].

A positive correlation between CRC progression and multi-antennary and sialylated glycans has been described in serum samples, in addition to a negative correlation between CRC progression and bi-antennary core-fucosylated N-glycans [99]. Finally, a downregulation of 23 N-glycan compositions (mostly galactosylated forms), in addition to an upregulation of mannose-rich HexNAc2Hex7, fucosylated bi-antennary glycan HexNAc4Hex5Fuc1NeuAc2, and tetra-antennary HexNAc6Hex7NeuAc3 was observed in the serum of CRC patients in stages II and III [100]. All biomarkers are summarized in Table 4.

**Table 4:** Main biomarkers found in blood samples of CRC patients with different omics technologies.

Omics	Biomarker	Change	Reference
Genomics	cfDNA	Increase	[80,81]
Genomics	*KRAS, APC, TP53*	Mutation	[78,82]
Genomics	cfDNA Microsatellite instability	Increase	[84,101]
Transcriptomics	*CK19, CK20, CEA, MDM2, DUSP6, CPEB4, MMD, EIF2S3, ANXA3, CLEC4D, LMNB1, PRRG4, TNFAIP6, VNN1,* and *IL2RB*	Upregulated	[76,78,85]
Transcriptomics	miR-145, miR-143, miR-135, miR-17-92, miR-92a, miR-29a, miR-125b, miR-19a-3p, miR-223–3p, miR-92a-3p and miR-422a, miR-21	Upregulated	[78,86,87]
Epigenomics	*SEPT9*	Methylation	[76]
Proteomics	CEA, CA19-9 and SAA	Increase	[76,88]
Proteomics	MST1/STK4 and S100A9	Increase	[83]
Proteomics	Cyr61	Increase	[89]
Proteomics	Antibodies against EDIL3, GTF2B, HCK, p53, PIM1 and STK4	Increase	[91]
Metabolomics	Glucose and long-chain hydroxy fatty acids	Decrease	[93,94]
Metabolomics	Pyruvic acid, lysine, glycolic acid, ornithine, fumaric acid	Increase	[96]
Metabolomics	Palmitoleic acid, tryptophan, lysine, 3-hydroxyisovaleric acid	Decrease	[96]
Glycomics	Galactosylation and sialylation of fucosylated IgG glycan structures	Decrease	[97]
Glycomics	Bisecting GlcNAc in IgG glycan structures	Increase	[97]
Glycomics	Glycans with no galactose residues, tri- and tetra-galactosylated glycans, tri- and tetra-sialyted structures, highly branched glycans	Increase	[98]
Glycomics	Mono- and di-galactosylated structures, mono-sialyted glycans, galactosylated and sialylated bi-antennary GlcNAc glycans, neutral core fucosylated glycans with one or two galactose residues	Decrease	[98]
Glycomics	Mannose-rich HexNAc2Hex7, fucosylated bi-antennary glycan HexNAc4Hex5Fuc1NeuAc2, tetra-antennary HexNAc6Hex7NeuAc3	Upregulated	[100]

### 3.5. Bowel Lavage Fluid Samples 

Before a colonoscopy, patients have to intake polyethylene glycol electrolyte lavage solutions to increase bowel visibility during the intervention. Once there, saline solution is applied directly to the area of the lesion to obtain the bowel lavage fluid (BLF), which contains a high concentration of cells in contact with this lesion. BLF presents some advantages in front of stool samples, such as lower bacterial interference and lower food leftovers, easier handling in the laboratory, less variability because of the different times in the bowel and water quantity and, finally, less protein degradation [102]. Nowadays, the use of BLF is not extended, although it is a very useful sample with high potential for new biomarkers that needs to be studied. 

#### 3.5.1. Genomics 

BLF genomics is based on DNA mutations and methylations, since DNA extraction is easier in BLF than in stool [103]. Twenty years ago, it was discovered that BLF from CRC patients presented an increase of mutated *KRAS* and *P53* [103,104]. Furthermore, BLF from CRC patients also presented mutations in *TGFβ RII* and *APC* genes [104]. The microbiome metagenomics in this type of sample has also been studied in CRC patients. There was an increase in *Proteobacteria* and *Fusobacteria* in BLF from CRC patients, as well as a decrease in *Firmicutes* [105]. In addition, Yuan et al. studied the difference between BLF and tumour tissue from CRC patients, demonstrating that *Proteobacteria*, *Firmicutes* and *Bacteroidetes* were increased in BLF in front of tumour tissue in these patients [106]. 

Regarding the epigenetics signature, Harada and collaborators analysed 14 targets for aberrant methylation of CpG islands in CRC by MethyLight assays with PCR. They found three possible biomarkers that allow the discrimination between CRC patients and healthy subjects that can be used individually or as a panel: miR-124-3, LOC386758, and SFRP1 [107]. Finally, the hypermethylation of SDC2, which has been mentioned before, has also been described in BLF [108]. All biomarkers are summarized in Table 5.

#### 3.5.2. Proteomics 

Proteomics displayed the worst results of all BLF biomarkers, since the identified proteins are not CRC-specific. For instance, Adnab-9 was found increased in patients with a high risk of CRC, but was also raised in coeliac patients’ stools [102]. Finally, haemoglobin was increased in BLF from CRC patients, but was also increased in patients with inflammatory bowel disease and diverticulosis [109]. 

#### 3.5.3. Microbiome Study

The use of selective media for *Bacteroides fragilis* for 3 days in BLF from CRC patients demonstrated that the identification of this species may serve as a CRC biomarker in this sample type [110], as shown in Table 5.

**Table 5:** Main biomarkers found in bowel lavage fluid samples of CRC patients with different omics technologies.

Omics	Biomarker	Change	Reference
Genomics	*KRAS, P53*	Mutation	[103,104]
Genomics	*TGFβ RII, APC*	Mutation	[104]
Genomics	miR-124-3, LOC386758, SFRP1	Methylation	[107]
Genomics	*SDC2*	Methylation	[108]
Genomics (metagenomics)	*Proteobacteria*, *Fusobacteria*	Increase	[105]
Genomics (metagenomics)	*Firmicutes*	Decrease	[105]
Microbiome study	*Bacteroides fragilis*	Presence	[110]

### 3.6. Tumour Tissue Samples

Tumour biopsies allow the direct study of the characteristics of the cancerous tissue. This information is of undoubted and often irreplaceable interest. Together with basic research, this has led to the development of the first general biomarkers for diagnosis, without forgetting, of course, anatomopathological studies. Currently, the diagnostic panel for CRC comprises MSI/mismatch repair (MMR) status, KRAS/NRAS, BRAF, and PIK3CA mutations, and HER2 amplification [111]. Commercial gene expression signatures for CRC have been developed and some are considered in NCCN and ESMO guidelines (reviewed in [112]). 

#### 3.6.1. Genomics

Lin et al. established that DNA damage response (DDR)-related ATM or BRCA2 somatic mutations are promising biomarkers for assessing the response of stage III CRC patients to oxaliplatin-based chemotherapy [113]. Very recently, in a bioinformatics analysis, Wills et al. conducted a whole genome-wide association study (GWAS) in a very large cohort of patients and reported an association with overall survival and rs79612564 in the receptor tyrosine kinase ERBB4. Patients with high ERBB4 expression in colon tumours showed worse survival; both the rs79612564 variant and ERBB4 were proposed as predictive biomarkers of survival [114]. Next generation sequencing (NGS), in addition to demonstrating that mutations are common in advanced colon tumours, has proven that tumours located in the right colon have more genetic aberrations than in the left colon. This could be responsible for the different responses of patients depending on the location of the tumour [115].

On the other hand, Van den Berg et al. have defined a methylation marker panel to distinguish between consensus molecular subtype 2 (canonical) and consensus molecular subtype 3 (metabolic) CRC (defined in [116]), which can be used to determine the patient’s treatment [117]. Finally, a 10-gene-methylation-based signature for prognosis prediction of CRC has also been established using the TCGA database and bioinformatics tools [118]. 

#### 3.6.2. Transcriptomics

Using bioinformatics analysis, a prognostic signature based on the expression of REG1B, TGM6, NTF4, PNMA5, and HOXC13 could provide significant prognostic value for CRC [119]. Gu et al. have identified and validated metastasis-associated biomarkers. Concretely, they described that FAS and GSR are downregulated, while CYP1B1 is overexpressed in CRC [120]. Another study showed that the prognosis of CRC was significantly correlated with the expression of the E-selectin gene (SELE) [121].

A prognostic signature comprising six autophagy-related lncRNAs was identified in patients with CRC and could be used for prognosis in these types of patients [122]. This signature includes AC125603.2, LINC00909, AC0 16876.1, MIR210HG, AC009237.14, and LINC01063 [122]. All biomarkers are summarized in Table 6. In a more complex study, Xi et al. performed a bioinformatic analysis to construct a competing endogenous RNA (ceRNA) network based on the differentially expressed lncRNA and RNAs in two colon cancer gene expression datasets [123]. In summary, they were able to identify two new regulatory pathways as LINC00114/miR-107/PCKS5, UCA1/miR-107/PCKS5, and UCA1/miR-129-5p/SEMA6A. Therefore, two new lncRNAs (LINC00114 and UCA1) were identified by bioinformatics analysis [123]. Furthermore, LINC00114 could be linked to the overall survival of colon cancer patients [123]. 

#### 3.6.3. Proteomics

In a study by Buttacavoli et al. performing a 2D-DIGE proteomic analysis on a paired tumour and normal adjacent tissues, transgelin (TAGL) was identified as a potential biomarker for CRC [124]. Using a similar design, performing a comparative proteomic and phosphoproteomic analysis of paired tumour and normal adjacent tissues, Vasaikar et al. identified an association between decreased CD8 T cell infiltration and increased glycolysis in MSI-H tumours, suggesting a shift to glycolysis in immune-resistant MSI-H tumours [125]. All biomarkers are summarized in Table 6.

#### 3.6.4. Glycomics

The study of glycomics in tumour tissue is characterized by the comparative between tumour tissue and non-tumour adjacent tissue. These studies demonstrate that there is a downregulation in the tumour tissue of glypican-3 and syndecan-1 [25], an under-representation of complex N-glycans and α2,3-sialylation [126], a decrease of bisecting GlNAcylation, Lewis-type fucosylation [127], 9 N-glycans (M/Z 973^2+^, 1055^2+^, 1060^2+^, 1075^2+^, 1162^2+^, 1177^2+^, 1264^2+^, 1279^2+^, 1352^2+^) [128], and a decrease of fucosylation levels and highly branched N-glycans in stage II CRC [129]. On the other hand, there is an increase in tumour tissue of glucosylceramide, lactosylceramide, monosialic acid ganglioside, globoside 4 [25], chondroitin sulphate, dermatan sulphate [130], high mannose, hybrid and paucimannosidic type N-glycans [126], α2,6-sialylation together with an increase in total sialylation in mid- to late tumours, mannose type N-glycan structures [127], glycan-Tn/STn-MUC1 [131], 3 N-glycans (M/Z 1013^2+^, 1116^2+^, 1228^2+^) [128], overrepresentation of oligomannosidic, bi-antennary hypogalactosylated and branched compositions [100], and an increase in stage II CRC of sialylation levels and high-mannose glycans [129]. All biomarkers are summarized in Table 6.

**Table 6:** Main biomarkers found in tissue samples of CRC patients with different omics technologies.

Omics	Biomarker	Change	Reference
Transcriptomics	CYP1B1	Upregulated	[120]
Transcriptomics	FAS, GSR	Downregulated	[120]
Transcriptomics	AC125603.2, LINC00909, AC0168676.1, MIR210HG, AC009237, LINC01063	Prognosis biomarkers	[122]
Proteomics	Transgelin	Decrease	[124]
Proteomics	CD8 T cell infiltration	Decrease	[125]
Proteomics	Glycolysis in MSI-H tumours	Increase	[125]
Glycomics	Glypican-3, syndecan-1	Downregulated	[25]
Glycomics	Glycosylceramide, lactosylceramide, monosialic acid ganglioside, globoside 4	Upregulated	[25]
Glycomics	Heparan sulphate	Decrease	[130]
Glycomics	Chondroitin sulphate, dermatan sulphate	Increase	[130]
Glycomics	Complex N-glycans, α2,3-sialylation	Decrease	[126]
Glycomics	High mannose, hybrid and paucimannosidic type N-glycans	Increase	[126]
Glycomics	Bisecting GlNAcylation, Lewis-Type fucosylation	Decrease	[127]
Glycomics	α2,6-sialylation, total sialylation, mannose type N-glycan structures	Increase	[127]
Glycomics	M/Z 973^2+^, 1055^2+^, 1060^2+^, 1075^2+^, 1162^2+^, 1177^2+^, 1264^2+^, 1279^2+^, 1352^2+^	Decrease	[128]
Glycomics	M/Z 1013^2+^, 1116^2+^, 1228^2+^	Increase	[128]
Glycomics	Fucosylation levels, highly branched N-glycans	Decrease	[129]
Glycomics	Sialylation, high-mannose glycans	Increase	[129]
Glycomics	Glycan-Tn/STn-MUC1	Increase	[131]
Glycomics	Oligomannosidic, bi-antennary hypogalactosylated, branched compositions	Increase	[100]

#### 3.6.5. Multi-Omics

The underlying factors of human disease are complex, and the multi-omics perspective is valuable in identifying the pathogenic factors of diseases [132]. There is a debate regarding the differences between left-sided colon cancer and right-sided colon cancer, which was studied with a multi-omics perspective by Hu et al. [132]. Gene mutation, DNA methylation, gene expression, and miRNA were integrally compared between left-sided and right-sided colon cancer datasets from TCGA [132]. The results suggest that there are more aggressive markers in the right-sided colon cancer with the activation of the phosphatidylinositol 3-kinase pathway (PI3K) pathway that shows crosstalk with the RAS and P53 pathways [132].

A multi-omics approach using a gene expression dataset, a miRNA-seq dataset, a copy number variation dataset, a DNA methylation dataset, and a transcription factor (TF) dataset was performed by Yang et al. and found that these types of approaches are more effective than the single omics dataset approach [133].

## 4. Use of Extracellular Vesicles as Colorectal Cancer Biomarkers

CRC cells release EVs since early stages. For this reason, the EVs’ cargo could be a possible molecular biomarker of early diagnosis and prognosis [5]. Minimal information for studies of extracellular vesicles (MISEV2018) defines EVs as “particles naturally released from the cell that are delimited by a lipid bilayer and cannot replicate” [134]. EVs present several advantages in front of other kinds of biomarkers. They are easy to get, and the samples of origin are not invasive. Furthermore, the lipid bilayer allows their stabilization in circulation and protects them from ribonucleases and DNases degradation. Finally, EVs are very abundant and possess a long half-life, and the DNA inside the EVs reflects the mutational state of tumours [6,135,136]. 

The EVs content is based on tumour cell-derived genome, transcriptome, and secretome. Concretely, the cargo are oncoproteins, transcriptional regulators, splicing factors, proteins related to the cytoskeleton, apoptosis, cell cycle, cellular signalling, oxidative stress, focal adhesions, cellular mobility, DNA fragments, RNA (mRNA, miRNAs, non-coding RNA), and suppressor tumoral mutated genes [135,137]. EVs could improve early CRC biomarkers, since they are released by tumoral cells and carry RNA, DNA, and proteins to target cells, participating in tumoral microenvironment, tumour formation, progression, angiogenesis, invasion, metastasis, chemoresistance, drug resistance, and recrudescence [5,135,137,138,139,140]. 

Nowadays, there is no standard technique for EVs isolation, which leads to differences in cargo, in addition to a lack of a standard classification [140]. The gold standard isolation technique is ultracentrifugation, but EVs can also be isolated by gradient centrifugation, microfiltration, polymer-based precipitation, size-exclusion chromatography, immunoaffinity chromatography, microfluidic filtering, commercial kits, or antibody immobilization against membrane proteins [5,135,140,141]. EVs cargo and function, in addition to the protection of the lipidic membrane, make them the future of CRC early diagnosis and prognosis.

## 5. Conclusions

**Figure 1:**
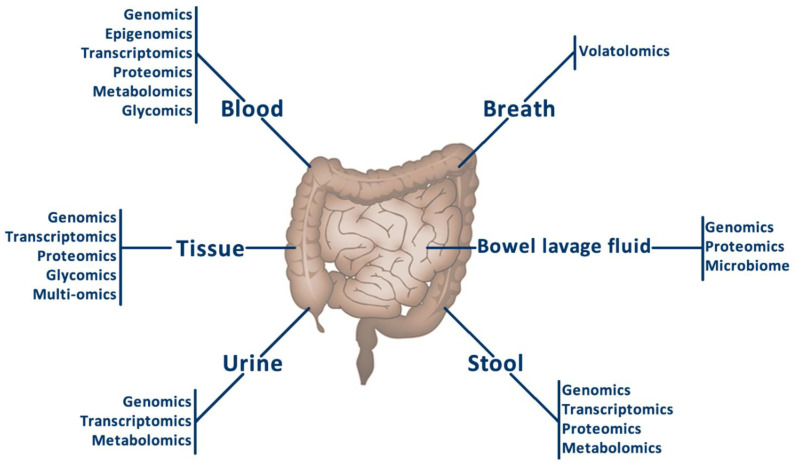
Main types of samples and the omics applied to them.

Omics techniques are a useful tool for new CRC biomarkers research, in both in situ tissue samples and different fluids related to this type of cancer. Great efforts and advances have been made by the scientific community to identify biomarkers through these techniques that could help in the management of CRC patients. The main types of samples and the omics applied to them are described in Figure 1. Despite the number of new biomarkers, there is a lack of standardization, since CRC is only diagnosed by colonoscopy, faecal occult blood testing, and the presence of CEA in plasma, although these techniques present some disadvantages. For these reasons, there is a need to study new sample types, such as bowel lavage fluid, and new biomarker source types, such as extracellular vesicles.

## Figures and Tables

**Table 1:** Main biomarkers found in breath samples of CRC patients with volatolomics.

Omics	Biomarker	Change	Reference
Volatolomics (GC-MS)	Benzaldehyde, Benzene ethyl, Indole	Upregulated	[30]
Volatolomics (GC-IMR-MS)	1,3-butadiene, N_2_O	Upregulated	[32]
Volatolomics (GC-IMR-MS)	Acetic acid, HNO_2_	Downregulated	[32]
Volatolomics (GC-MS)	1,3,5-cycloheptatriene	Upregulated	[40]
Volatolomics (GC-MS)	Tetradecane, Ethylbenzene, Methylbenzene, 5,9-Undecadien-2-one, 6,10-dimethyl, Benzaldehyde, Decane, Benzoic acid, 1,3-Bis(1-methylethenyl) benzene, Dodecane, Ethanone, 1[4-(1-methylethenyl)phenyl], acetic acid	Upregulated	[40,41]
Volatolomics (GC-MS)	Decanal, 2-Ethyl-1-hexanol	Downregulated	[40]
